# MEX3A is upregulated in esophageal squamous cell carcinoma (ESCC) and promotes development and progression of ESCC through targeting CDK6

**DOI:** 10.18632/aging.103196

**Published:** 2020-11-14

**Authors:** Lei Wei, Bo Wang, Liwen Hu, Yang Xu, Zhongdong Li, Yi Shen, Hairong Huang

**Affiliations:** 1 Department of Cardiothoracic Surgery, Jinling Hospital, Nanjing 210002, China; 2 Department of Thoracic Surgery, Nanjing Chest Hospital, Nanjing 210029, China

**Keywords:** esophageal squamous cell carcinoma, MEX3A, CDK6, cell proliferation, cell apoptosis

## Abstract

Esophageal squamous cell carcinoma (ESCC) is one of the most commonly diagnosed malignant tumors worldwide and identified as a serious threat to human health. The role of MEX3A in ESCC remains unclear. In this study, we found that MEX3A was upregulated in tumor tissues of ESCC and positively associated with more advanced tumor stage, higher risk of lymphatic metastasis and poor prognosis. The downregulation of MEX3A in ESCC cell lines could induce inhibition of cell proliferation, colony formation, cell migration, and the promotion of cell apoptosis, while MEX3A overexpression exhibited opposite effects. *In vivo* experiments also verified the inhibition of ESCC induced by MEX3A knockdown. Moreover, we identified CDK6 as a potential target of MEX3A, which was also upregulated in ESCC. Further studies demonstrated that knockdown of CDK6 showed similar effects on the development of ESCC with MEX3A. More importantly, it was illustrated that CDK6 knockdown could alleviate the promotion effects of MEX3A overexpression on ESCC. In conclusion, MEX3A was identified as a tumor promotor in the development and progression of ESCC by targeting CDK6, which may be considered as a novel prognostic indicator and therapeutic target in treatment of ESCC.

## INTRODUCTION

Esophageal cancer is a common malignant tumor that seriously endangers human health. According to histological classification, more than 90% of esophageal tumors can be classified as esophageal squamous cell carcinoma (ESCC) or esophageal adenocarcinoma [1, 2]. According to statistical data, nearly 500,000 new cases of ESCC are diagnosed annually worldwide, and the morbidity is rapidly increasing [3, 4]. Because of the unspecific symptoms and the lack of early diagnostic technologies, more than 50% of the patients with ESCC were diagnosed in advanced stage and could not undergo surgical resection. Therefore, the 5-year survival rate of ESCC is below 20%, and the prognosis is very poor [5–7]. In recent years, although the diagnostic and therapeutic methods for ESCC have been developed continuously, the mortality rate has not been significantly improved [8, 9]. Moreover, molecular targeted therapy which have achieved good therapeutic effects in many tumors such as hepatocellular carcinoma, colorectal cancer, breast cancer and so on have not yet played its role in the treatment of ESCC [10–17], which is resulted from the unclear molecular mechanism of ESCC. Therefore, the study of molecular mechanism of the occurrence, development and metastasis of ESCC is of great significance to establish effective targeted therapy, so as to improve the cure rate and reduce mortality of ESCC.

MEX3 protein was first discovered by Draper et al. in *Caenorhabditis elegans*. It is a translation regulator that can inhibit Pal-1 translation in early embryos of *Caenorhabditis elegans* [18]. MEX3 protein is a RNA-bound phosphoprotein, with two tandem repetitive heterogenous ribonucleoprotein K homologous (KH) domains at the N-terminal and a ring finger domain at the C-terminal. Through the KH domain, MEX3 binds to mRNA and shuttles between nucleus and cytoplasm through CRM1-dependent pathway, thus translating, regulating and degrading mRNA. Human MEX3 protein may mediate ubiquitination of target protein through ring finger domain, thus regulating its stability and subcellular localization [19–21]. As one of the MEX3 homologous genes, MEX3A also participates in the regulation of mRNA and may mediate the occurrence and development of many diseases, such as cancer. Till now, the role of MEX3A has been elucidated in several types of human cancer including gastric cancer, colorectal cancer and nephroblastoma [22–24]. However, the association between MEX3A and ESCC is still unclear.

In this study, we found that MEX3A was significantly upregulated in tumor tissues of ESCC, which was also verified in ESCC cell lines. High expression of MEX3A was found to be correlated with more advanced tumor stage, higher risk of lymphatic metastasis and poor prognosis. *In vitro* experiments exhibited that knockdown/overexpression of MEX3A in ESCC cells could significantly inhibit/promote cell proliferation, colony formation, migration and promote/suppress cell apoptosis. The inhibition of tumor growth *in vivo* was also demonstrated by construction of mice xenograft model. Moreover, we found that MEX3A may execute its effects on ESCC through inducing the expression of CDK6. These findings suggested the potential role of MEX3A in ESCC as a tumor promotor, which may be used as a prognostic indicator and therapeutic target in development of targeted therapy.

## RESULTS

### MEX3A was upregulated in ESCC and correlated with poor prognosis

**Figure 1:**
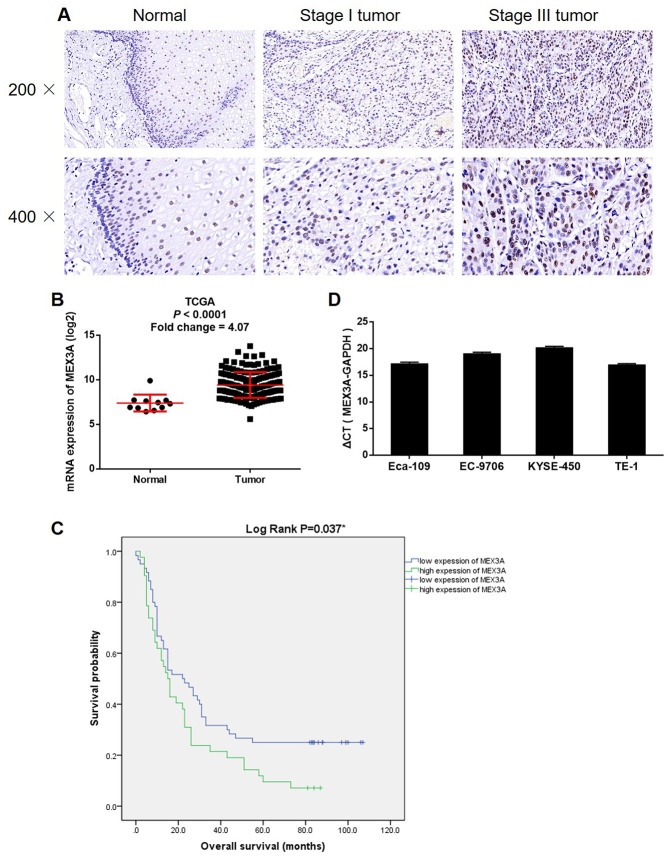
**MEX3A was upregulated in ESCC.** (**A**) The expression of MEX3A in ESCC tumor tissues and normal tissues was detected by IHC. (**B**) The expression profiling data collected from TCGA showed that MEX3A was upregulated in ESCC. (**C**) Kaplan-Meier survival analysis was performed to reveal the relationship between MEX3A expression and prognosis of ESCC patients. (**D**) The background expression of MEX3A in ESCC cell lines including Eca-109, EC-9706, KYSE-450 and TE-1 was detected b qPCR.

**Figure 2:**
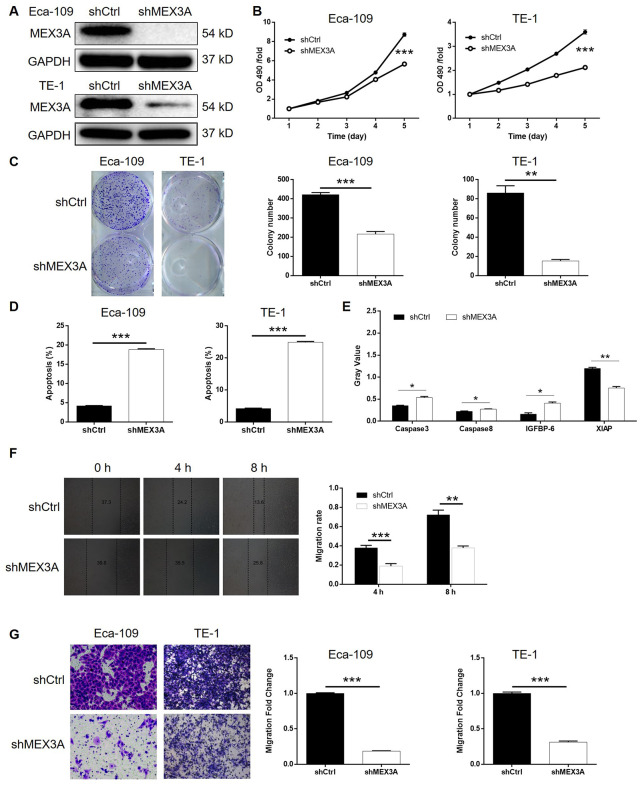
**MEX3A knockdown inhibited development of ESCC *in vitro*.** (**A**) The knockdown of MEX3A in Eca-109 and TE-1 cells was verified by western blotting. (**B**) MTT assay was performed to examine the effects of MEX3A knockdown on cell proliferation of Eca-109 and TE-1 cells. (**C**) The effects of MEX3A knockdown on the ability of Eca-109 and TE-1 cells to form colonies were evaluated by colony formation assay. (**D**) Flow cytometry was used to detect cell apoptosis of Eca-109 and TE-1 cells cells with or without MEX3A knockdown. (**E**) The differentially expressed apoptosis related proteins identified in Eca-109 cells with or without MEX3A knockdown by Human Apoptosis Antibody Array. (**F**, **G**) Cell migration ability of Eca-109 and TE-1 cells with or without MEX3A knockdown was detected by wound healing assay (**F**) and Transwell assay (**G**). The figures are representative data from at least three independent experiments. The data were expressed as mean ± SD (n ≥ 3), *P<0.05, **P<0.01, ***P<0.001.

For investigating the role played by MEX3A in ESCC, IHC was performed to detect the expression of MEX3A in clinical specimens collected from 103 patients with ESCC. It was demonstrated that MEX3A expression was significantly elevated in tumor tissues compared with normal tissues (*P* < 0.001, Figure 1A and Table 1). The statistical analysis of the relationship between MEX3A expression with tumor characteristics showed that high MEX3A expression was associated with advanced tumor stage and higher risk of lymphatic metastasis (*P* < 0.01, Table 2), which was further verified by Spearman rank correlation analysis (*P* < 0.01, Supplementary Table 3). Consistently, the gene expression profiling data collected from The Cancer Genome Atlas (TCGA) also showed the upregulation of MEX3A in ESCC tumor tissues (Fold change 4.07, *P* < 0.001, Figure 1B). More importantly, the Kaplan-Meier survival analysis revealed that high expression of MEX3A was correlated with poor prognosis (Log rank *P* < 0.05, Figure 1C). Besides, background expression of MEX3A in ESCC cell lines was detected by qPCR, which showed high expression in Eca-109, EC-9706, KYSE-450 and TE-1 cells (Figure 1C). Therefore, Eca-109 and TE-1 cells were transfected with lentivirus expressing shCtrl or shMEX3A to construct MEX3A knockdown cell models. Through detecting the fluorescence, transfection efficiency was assured to be >80% in both cell lines (Supplementary Figure 1A). We also showed 80.1% and 51.7% knockdown of MEX3A in Eca-109 and TE-1 cells by qPCR (*P* < 0.01, Supplementary Figure 1B), respectively, which was further confirmed by western blotting (Figure 2A). Collectively, the upregulated expression of MEX3A in ESCC was illustrated and MEX3A knockdown cell models were constructed for further studies.

**Table 1:** Expression patterns of MEX3A in ESCC tissues and normal tissues revealed in immunohistochemistry analysis.

**MEX3A expression**	**Tumor tissue**		**Normal tissue**	
	**Cases**	**Percentage**	**Cases**	**Percentage**
Low	61	59.2%	66	100%
High	42	40.8%	0	-

*P* < 0.001.

**Table 2:** Relationship between MEX3A expression and tumor characteristics in patients with ESCC.

**Features**	**No. of patients**	**MEX3A expression**		***P* value**
		**low**	**high**	
All patients	103	61	42	
Age (years)				0.877
≤ 65	53	31	22	
> 65	50	30	20	
Gender				0.069
Male	76	41	35	
Female	27	20	7	
Lymph node positive				0.003**
< 1	38	30	8	
≥ 1	54	26	28	
Tumor size				0.317
< 5 cm	41	25	16	
≥ 5 cm	42	21	21	
Grade				0.504
I	8	6	2	
II	72	42	30	
III	23	13	10	
AJCC Stage				0.004**
1	4	3	1	
2	43	32	11	
3	51	23	28	
T Infiltrate				0.862
T1	4	3	1	
T2	12	6	6	
T3	81	49	32	
T4	3	1	2	
Lymphatic metastasis (N)				0.005**
N0	46	34	12	
N1	31	16	15	
N2	19	10	9	
N3	4	0	4	

### MEX3A knockdown inhibited development of ESCC *in vitro*

Given the successful construction of MEX3A knockdown cell models, we next investigated its role in ESCC *in vitro*. The results of MTT assay showed that, after knockdown of MEX3A, cell proliferation was decreased in Eca-109 and TE-1 cells (*P* < 0.001, Figure 2B). It was also demonstrated that, in shMEX3A group, obviously less colonies were formed compared with shCtrl group after 14 days of Eca-109 and TE-1 cell culture, respectively (*P* < 0.01, Figure 2C). The detection of cell apoptosis by flow cytometry showed apparently increased apoptotic cell percentage in shMEX3A group compared with shCtrl group of Eca-109 and TE-1 cells, respectively, indicating the ability of MEX3A knockdown to induce cell apoptosis (*P* < 0.001, Supplementary Figure 2A and 2D). Moreover, the effects of MEX3A knockdown on apoptosis related proteins were screened by a Human Apoptosis Antibody Array based on TE-1 cells with or without MEX3A knockdown (Supplementary Figure 2B). The results indicated that MEX3A knockdown caused the upregulation of Caspase3, Caspase8 and IGFBP-6, and downregulation of XIAP (*P* < 0.05, Figure 2E). Furthermore, the effects of MEX3A knockdown on cell motility were evaluated by wound healing and Transwell assays simultaneously. As shown in Figure 2F, upon MEX3A knockdown, cell migration rate was significantly decreased in TE-1 cells (*P* < 0.01). Similar results were also obtained from Transwell assay (*P* < 0.001, Figure 2G). In summary, these results suggested that knockdown of MEX3A could inhibit the development of ESCC *in vitro*.

### MEX3A knockdown inhibited tumor growth of ESCC *in vivo*

**Figure 3:**
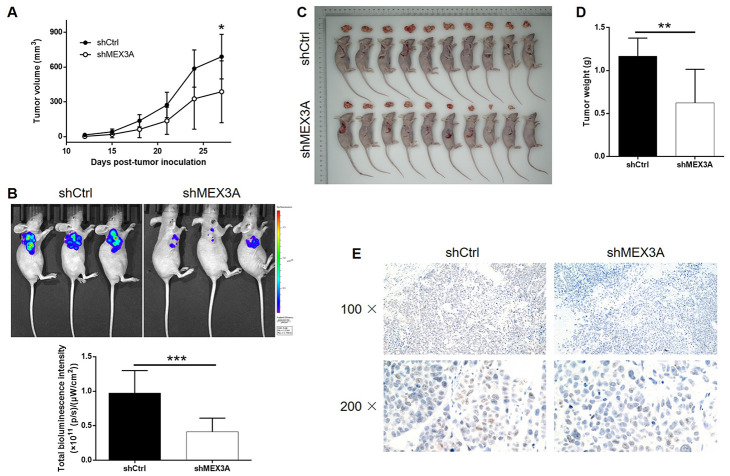
**MEX3A knockdown inhibited development of ESCC *in vivo*.** Mice xenograft models were constructed through subcutaneously injecting Eca-109 cells with or without MEX3A knockdown. (**A**) The volume of tumors on mice was measured and calculated at indicated intervals during the culture of the animal models. (**B**) *In vivo* imaging of the tumors on mice was facilitated by the injection of D-luciferin before sacrificing the mice. (**C**) The photos of the tumors removed from the sacrificed mice were collected by a digital camera. (**D**) Tumor weight was measured after obtaining the removed tumors from animal models. (**E**) Tumor sections obtained from the removed tumors were subjected to IHC analysis to detect the expression of Ki-67. The data were expressed as mean ± SD (n ≥ 3), *P<0.05, **P<0.01, ***P<0.001.

In order to further explore the effects of MEX3A on development of ESCC *in vivo*, mice xenograft models were established through injection of TE-1 cells with or without MEX3A knockdown. Tumor volume was measured throughout the culture of animal models, which showed significantly slower growth of tumors in shMEX3A group (*P* < 0.05, Figure 3A). Before sacrificing the mice, *in vivo* imaging of tumors was facilitated by injection of D-Luciferin and showed obviously weaker luminescence intensity, as well as smaller tumor, in shMEX3A group (*P* < 0.001, Figure 3B). Following the sacrifice of animal models, the photos of the tumors were collected (Figure 3C) and weights were measured (*P* < 0.01, Figure 3D), both indicating smaller tumors in shMEX3A group. Moreover, the Ki-67 expression in sections of tumors collected from both groups was assessed by IHC and demonstrated significantly lower expression in shMEX3A group compared with shCtrl group (Figure 3E). All the above results suggested that knockdown of MEX3A could suppress tumor growth of ESCC *in vivo*.

### The exploration of downstream mechanism of MEX3A-induced regulation of ESCC

**Figure 4:**
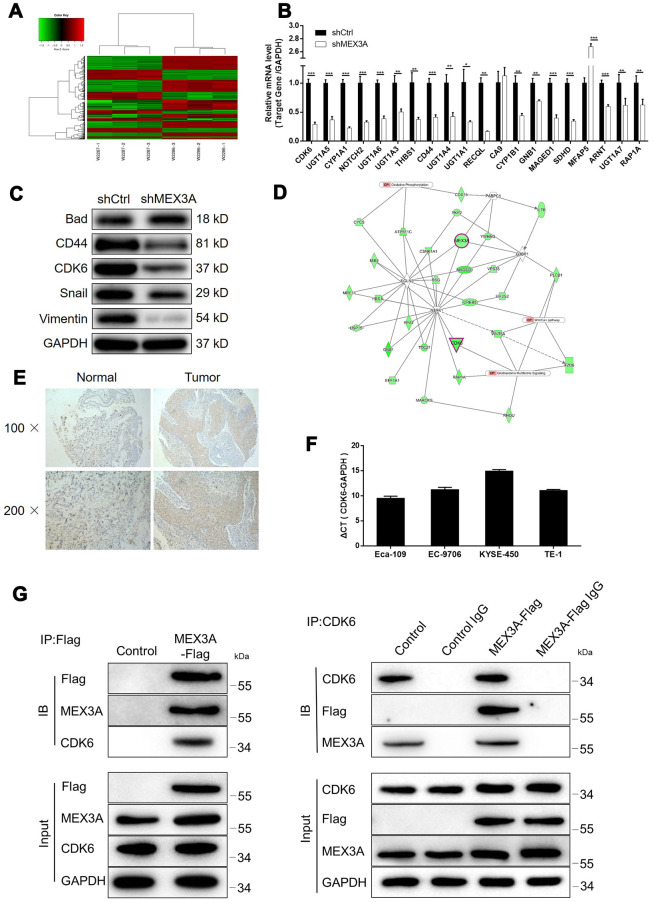
**MEX3A knockdown may inhibit ESCC through targeting CDK6.** (**A**) Heatmap of the RNA sequencing performed on Eca-109 cells with or without MEX3A knockdown (3 v 3). (**B**) The mRNA expression of several most significantly down-regulated DEGs in shMEX3A group identified by RNA sequencing was further detected in TE-1 cells. (**C**) The protein levels of various down-regulated DEGs in shMEX3A groups were verified in TE-1 cells through western blotting. (**D**) The MEX3A-related interaction network was predicted based on IPA analysis. (**E**) The expression of CDK6 in ESCC tumor tissues and normal tissues was detected and compared by IHC analysis. (**F**) The background expression of CDK6 in ESCC cell lines including Eca-109, EC-9706, KYSE-450 and TE-1 was detected by qPCR. (**G**) Co-IP assay was performed to verify the direct interaction between MEX3A and CDK6. The data were expressed as mean ± SD (n ≥ 3), *P<0.05, **P<0.01, ***P<0.001.

For exploring the mechanism underlying the inhibition of ESCC by MEX3A knockdown, RNA sequencing was performed to obtain the gene expression profiling in TE-1 cells with or without MEX3A knockdown (3 v 3). In total, 314 differentially expressed genes (DEGs) were identified, among which 117 were upregulated and 197 were downregulated (Figure 4A and Supplementary Figure 3A, 3B). Subsequently, IPA analysis based on all the DEGs revealed the most enriched canonical signaling pathway (Supplementary Figure 3C). The analysis of IPA disease & function indicated that the most favorable one regulated by MEX3A was cancer (Supplementary Figure 3D). Moreover, several most downregulated DEGs in shMEX3A group were subjected to qPCR and western blotting for detecting their mRNA or protein levels in Eca-109 cells, which identified CDK6 as a promising downstream of MEX3A (*P* < 0.05, Figure 4B and 4C). Further combining the the analysis of MEX3A-related interaction network, we supposed that MEX3A knockdown may execute its function in ESCC through downregulating CDK6 (Figure 4D). The following detection of CDK6 in ESCC tissues and normal tissues confirmed this speculation by showing significant downregulation of CDK6 in ESCC (Figure 4E). The background expression of CDK6 in ESCC cell lines was further detected by qPCR and also showed high expression in Eca-109, EC-9706, KYSE-450 and TE-1 cells (Figure 4F). More importantly, our preliminary investigation of the underlying mechanism between MEX3A and CDK6 by co-IP assay revealed the direct interaction between them (Figure 4G). All the above results identified CDK6 as a potential downstream of MEX3A in the promotion of ESCC.

### Knockdown of CDK6 attenuated the effects of MEX3A overexpression on ESCC

**Figure 5:**
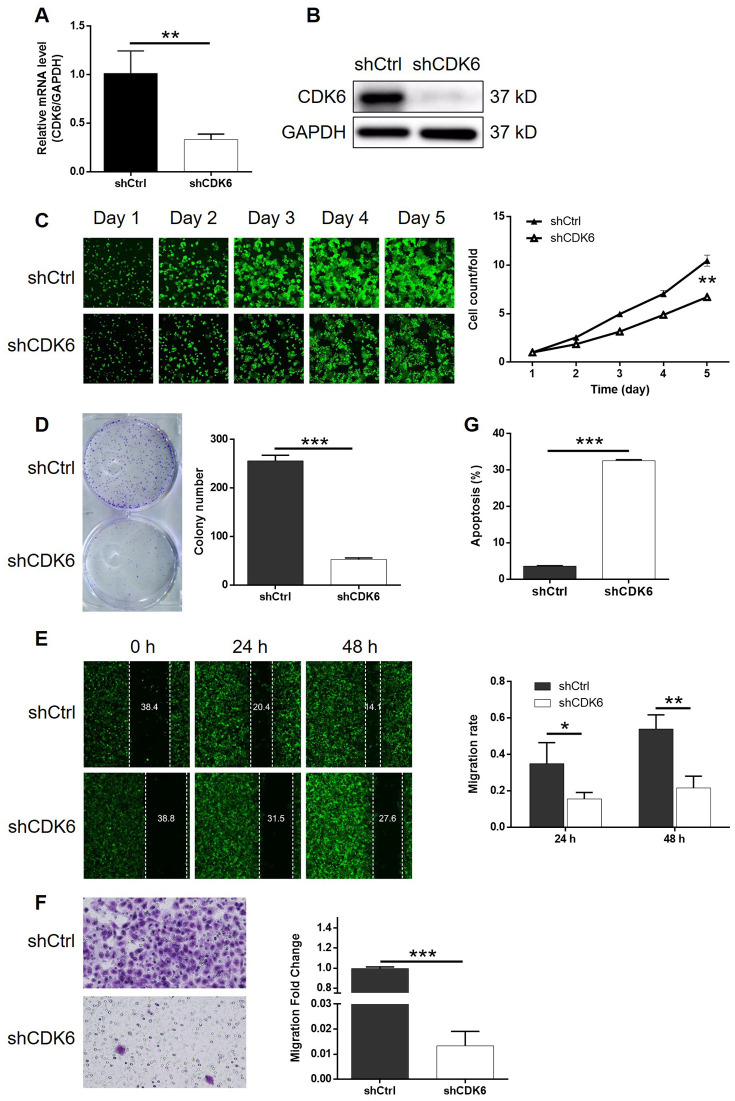
**Knockdown of CDK6 inhibited development of ESCC *in vitro*.** (**A**) The efficiency of CDK6 knockdown in Eca-109 cells was detected by qPCR. (**B**) The knockdown of CDK6 in Eca-109 cells was confirmed by western blotting. (**C**) The inhibition of cell proliferation by CDK6 knockdown in Eca-109 cells was evaluated by Celigo cell counting assay. (**D**) The influence of colony formation ability of Eca-109 cells by CDK6 knockdown was examined by colony formation assay. (**E**, **F**) The effects of CDK6 knockdown on cell migration of Eca-109 cells were assessed by wound-healing (**E**) and Transwell (**F**) assays. (**G**) Flow cytometry was performed to detect cell apoptosis of Eca-109 cells with or without CDK6 knockdown. The figures are representative data from at least three independent experiments. The data were expressed as mean ± SD (n ≥ 3), *P<0.05, **P<0.01, ***P<0.001.

**Figure 6:**
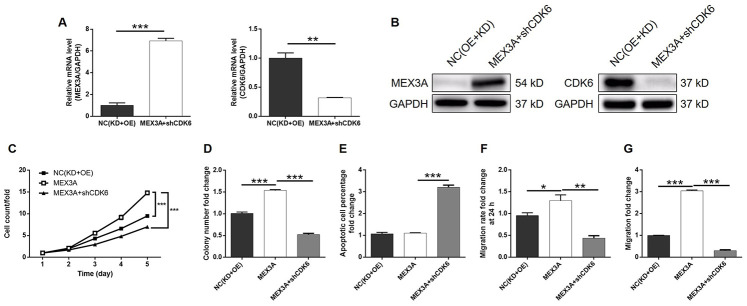
**CDK6 knockdown alleviated the effects of MEX3A overexpression on ESCC.** (**A**) The expression of MEX3A and CDK6 in Eca-109 cells with simultaneous MEX3A overexpression and CDK6 knockdown was detected by qPCR and compared with negative control. (**B**) The change of expression of MEX3A and CDK6 in MEX3A+shCDK6 group of Eca-109 cells was visualized by western blotting. (**C**–**F**) The results of Celigo cell counting assay (**C**), colony formation assay (**D**), flow cytometry (**E**), wound-healing assay (**F**) and Transwell assay (**G**) showed that the effects of MEX3A overexpression on cell proliferation, colony formation, cell apoptosis and cell migration could be alleviated by CDK6 knockdown.

In order to investigate the synergistic effect of MEX3A and CDK6 on the development of ESCC, Eca-109 cells with MEX3A overexpression (MEX3A group), CDK6 knockdown (shCDK6 group) and simultaneous MEX3A overexpression and CDK6 knockdown (MEX3A+shCDK6 group) were constructed and verified using abovementioned method (Supplementary Figure 4A). Following the verification of CDK6 knockdown by qPCR (67.3% knockdown) and western blotting (*P* < 0.01, Figure 5A and 5B), detection of cellular functions demonstrated 35.8%, 79.3%, 59.9% (wound-healing assay) and 98.7% (Transwell assay) inhibition of cell proliferation, colony formation and cell migration, respectively (*P* < 0.01, Supplementary Figures 4B and 5C–5F), as well as 9.0-fold increase of cell apoptosis (*P* < 0.001, Figure 5G) in shCDK6 group. These results suggested a similar role of CDK6 in ESCC with MEX3A. Conversely, MEX3A overexpression induced 1.56-fold, 1.52-fold and 1.32-fold (wound-healing assay) and 3.05-fold (Transwell assay) elevation of cell proliferation, colony formation and cell migration (*P* < 0.05, Supplementary Figure 5A–5F), however showing ignorable effects on cell apoptosis (Supplementary Figure 5G). More importantly, we found that, with upregulated expression of MEX3A and downregulated expression of CDK6 in MEX3A+shCDK6 group (*P* < 0.01, Figure 6A and 6B), the effects of MEX3A overexpression on cell proliferation, colony formation, cell apoptosis and cell migration could be significantly alleviated even reversed by CDK6 knockdown (*P* < 0.05, Supplementary Figure 6 and Figure 6C–6G).

## DISCUSSION

Esophageal cancer is the eighth most common cancer worldwide and one of the most common malignant tumors in China [1, 2]. ESCC is the most common histological type of esophageal cancer [1]. Despite the continuous improvement in the methods of comprehensive treatment such as surgery, radiotherapy and chemotherapy, the prognosis and 5-year survival rate of patients with ESCC remain poor. Targeted therapy for ESCC inhibits the proliferation and migration of cancer cells and promotes apoptosis by regulating related target or signaling pathway [25–28]. Up to now, a variety of molecular targets and related regulatory mechanisms of ESCC have been discovered and various targeted therapeutic drugs have been designed, some of which have achieve certain effects in clinical trials [29–34]. Meanwhile, the exploration of novel targets is still in continuous progression so as to improve the therapeutic effects against ESCC [35–38].

As one of the MEX3 homologous genes, MEX3A participates in the regulation of mRNA and may be involved in the occurrence and development of many diseases, including cancer. Jiang et al. [22] found that knockdown of MEX3A in gastric cancer cells could effectively inhibit the proliferation of cancer cells and colony formation, indicating that MEX3A was involved in cell transformation. In addition, wound healing assay and Transwell assays showed that MEX3A knockdown significantly inhibited the migration of gastric cancer cells. The significantly upregulated expression in gastric cancer tissues compared with normal tissues also indicated MEX3A as a participant in development and progression of gastric cancer. Pereira et al. speculated that MEX3A participated in the regulation of CDX2 and confirmed that MEX3A inhibited the expression of CDX2 and damaged the differentiation of intestinal cells, suggesting that MEX3A may be an oncogene for colorectal cancer [23]. Moreover, Krepischi et al. found that MEX3A was overexpressed in nephroblastoma by microarray comparative genomic hybridization (Array-CGH) [24]. Despite of these studies, the relationship between MEX3A and breast cancer is still unclear.

In this study, we found that expression of MEX3A was significantly upregulated in ESCC tumor tissues compared with normal tissues, which was in consistent with data mining of TCGA. Moreover, MEX3A high expression was positively associated with advanced tumor stage, higher risk of lymphatic metastasis and shorter survival period. The following *in vitro* experiments elucidated that knockdown of MEX3A significantly inhibited cell proliferation, colony formation and cell migration, while promoting cell apoptosis of ESCC cells. Meanwhile, MEX3A overexpression exhibited conversed effects against MEX3A knockdown, emphasizing the role of MEX3A in ESCC as a tumor promotor. More importantly, the ability of MEX3A knockdown to restrain tumor growth *in vivo* suggested that it may be used as novel therapeutic target in the treatment of ESCC. Furthermore, we found the upregulation of pro-apoptotic proteins Caspase3, Caspase8 and IGFBP-6, and downregulation of anti-apoptotic protein XIAP in ESCC cells with MEX3A knockdown, by which may MEX3A suppresses cell apoptosis of ESCC cells.

CDK6 belongs to the CDC-2 related kinase family and has serine/threonine kinase activity. It is a key cell cycle regulator in mammalian cells and encoded by *PLSTIRE* in human. Accumulating evidence showed that overexpression or increased activity of CDK6 existed in various types of malignant tumors, which could also act as mediator in the promotion of malignant tumors by other molecules. For example, Xue et al. identified CDK6 as a key mediator in the promotion effects of hsa_circ_0081143/miR-646 axis on the development and drug resistance of gastric cancer [39]. Wang et al. reported that the CDK6 inhibitor PD0332991 could significantly inhibit cell proliferation of gastric cancer *via* the modulation of cell cycle [40]. In non-small cell lung cancer (NSCLC), CDK6 was also identified as a key factor in the ceRNA mechanism of lncRNA HNF1A-AS1/miR-149, whose downregulation suppressed the tumorigenic ability of NSCLC [41]. Notably, Baba et al. analyzed the expression of CDK6 in 129 ESCC samples by array comparative genomic hybridization analysis. The results showed that there was abnormal amplification of CDK6 in ESCC samples, and the survival period of ESCC patients with high expression of CDK6 was significantly shorter than that of ESCC patients with low expression of CDK6, indicating that CDK6 played an oncogene role in ESCC [42].

In this study, through RNA sequencing, CDK6 was identified as the potential downstream in the MEX3A induced promotion of ESCC, which has direct interaction with MEX3A. Further verification revealed that, similar with MEX3A, CDK6 was also upregulated in ESCC tumor tissues compared with normal tissues, which was also in consistent with the previous report [42]. The following *in vitro* experiments using CDK6 knockdown ESCC cell lines also proved the inhibition of ESCC development by CDK6 knockdown, represented by suppression of cell proliferation, colony formation, cell migration and promotion of cell apoptosis. More importantly, we further illustrated that knockdown of CDK6 could alleviate or reverse the promotion effects of MEX3A overexpression on ESCC, indicating its role as the downstream of CDK6 again.

In conclusion, our study identified MEX3A as a tumor promotor in ESCC, which may be used as a prognostic indicator and novel therapeutic target in the treatment of ESCC. We found that MEX3A was upregulated in ESCC tumor tissues and its upregulation or downregulation could promote or suppress the development and progression of ESCC. Moreover, our results provided evidence that MEX3A may execute its effects on ESCC through upregulating CDK6.

## MATERIALS AND METHODS

### Immunohistochemistry

Human ESCC and normal formalin fixed and paraffin embedded tissue microarray was purchased from Shanghai Outdo Biotech Co., Ltd. (Shanghai, China). Patient information data were collected. Written informed consent was provided by each patient. Our study protocol was approved by Ethics committee of Jinling Hospital. Before immunohistochemistry analysis, the microarray was baked at 51°C for 30 min. Antigen retrieval was performed using citric acid buffer at 180°C for 15 min and the endogenous peroxidase was blocked with 3% H_2_O_2_ for 5 min. All slices were incubated with rabbit anti-MEX3A at 4°C overnight. After washing, the second antibody was added and incubated for 2 h at room temperature. MEX3A staining was developed with diaminobenzene and hematoxylin. Images were acquired with microscopic and viewed with ImageScope software. All slides were examined randomly by two independent pathologists. Staining percentage scores were classified as: 1 (1%-24%), 2 (25%-49%), 3 (50%-74%) and 4 (75%-100%). Staining intensity were scored 0 (Signalless color) to 3 (light yellow, brown and dark brown). IHC outcomes were determined by percentage and intensity scores.

### Cell culture

Eca-109 cells were cultured in CM1-1 medium, containing 90% DMEM-H and 10% fetal bovine serum, TE-1 were cultured in CM1-1 medium, containing 90% RPMI 1640 medium supplemented with 10% fetal bovine serum. EC-9706 and KYSE-450 purchased from American Type Culture Collection (ATCC) (Manassas, VA, USA) and maintained in Dulbecco’s Modified Eagle’s Medium. TE-1 and Eca-109 were purchased from BeNa Technology (Hangzhou, Zhejiang, China). All cell lines grow at 37°C in the presence of 5% CO_2_. All medium was renewal every 2 to 3 days.

### Plasmid construction, lentivirus infection and cell transfection

The overexpressing MEX3A construct and knockdown sequences (5’-AGGCAAGGCTGCAAGATTAAG-3’) of MEX3A gene and knockdown sequences (5’-AAGGATATGATGTTTCAGCTT-3’, 5’-TGGCTGCATATTTGCAGAAAT-3’, 5’-GCCCAACCAATTGAGAAGTTT-3’) of CDK6 gene were designed by Shanghai Bioscienceres (Shanghai, China). These sequences were annealed and ligated into the retroviral BR-V-108 or BR-V112 vector. And plasmids were extract by EndoFree maxi plasmid kit (Tiangen, Beijing, China). Retrovirus packaging and infection were conducted subsequently.

Eca-109, EC-9706, KYSE-450, TE-1 cells were transfected with 20 μL 1×10^8^ TU/mL Lentivirus vectors. Cells grow in culture medium with Enhanced Infection Solution (ENI.S, pH = 7.4) and Polybrene at 37°C in the presence of 5% CO_2_ for 72 h. Fluorescence expression was observed under fluorescence microscope, and fluorescence intensity was valued.

### RNA extraction and RT-qPCR

Total RNA was extracted from fully lysed cells using TRIzol reagent (Sigma, St. Louis, MO, USA). The quality of total RNA was evaluated by Nanodrop 2000/2000C spectrophotometer (Thermo Fisher Scientific, Waltham, MA, USA) according to the manufacturer’s instructions. 2.0 μg total RNA was reverse transcribed using Promega M-MLV Kit (Promega, Heidelberg, Germany) and quantitative real-time PCR was performed on Biosystems 7500 Sequence Detection system applied with Vazyme SYBR Green qPCR Kit (Nangjing, Jiangsu, China). GAPDH was acted as control, and the related primers used for the PCR reaction showed in Supplementary Table 1. Reactions were performed in triplicate and the relative quantitative analysis in gene expression data were analyzed by the 2^−ΔΔCt^ method.

### Western blotting

Eca-109 and TE-1 Cells were lysed in ice-cold RIPA buffer (Millipore, Temecula, CA, USA) and the total protein concentration was detected by a BCA Protein Assay Kit (HyClone-Pierce, Logan, UT, USA). 20 μg proteins were separated by 10% SDS-PAGE (Invitrogen, Carlsbad, CA, USA) and electrophoretically transferred onto polyvinylidene fluoride membranes. The membranes were blocked in 5% nonfat milk Tween-Tris-buffer for 2 h at room temperature and then incubated with primary antibodies (antibodies used were detailed in Supplementary Table 2). Then membranes were continuing incubated with HRP secondary antibody. The blots were visualized by enhanced chemiluminescence (ECL) (Amersham, Chicago, IL, USA).

### MTT assay

Lentivirus transfected Eca-109 and TE-1 cells were seeded into a 96-well plate (3,000 cells/well) in triplicate. 20 μL MTT solution (5 mg/mL, GenView, El Monte, CA, USA) was added and incubated for 4 h and then 100 μL DMSO solution was added. The absorbance values at 490 nm were measured by microplate reader (Tecan, Männedorf, Zürich, Switzerland) and the reference wavelength was 570 nm. The cell viability ratio was calculated according to the equation: Cell viability (%) = optical density (OD) treated/OD control × 100%.

### Flow cytometry for apoptosis

Lentivirus transfected Eca-109 and TE-1 cells were inoculated in 6-well plates with 2 mL per well at a seeding density of 800 cells/mL in triplicate and further cultured for 5 days. Floating cells were washed with 4°C ice-cold D-Hanks twice. Then we stained the cells with 5μL Annexin V-APC (eBioscience, San Diego, CA, USA) without light. Apoptosis analyses was measured using FACSCalibur (BD Biosciences, San Jose, CA, USA).

### Colony formation assay

Lentivirus transfected Eca-109 and TE-1 cells in the logarithmic growth phase were trypsinized, resuspended and seeded into 6-well plates (500 or 1000 cells/well) in triplicate and further cultured in the RPMI-1640 medium with 10% FBS. The culture medium was exchanged every 72 h. Cell clones were photographed under a fluorescence microscope (Olympus, Tokyo, Japan). 1 mL 4% paraformaldehyde were added to fix cells and 500 μL of Giemsa were added for cell staining. Then the cells were washed and dried for photograph with a digital camera. Colony forming rate = (colony number/inoculated cell number) × 100%.

### Transwell assay

Successfully transfected Eca-109 and TE-1 cells were incubated in the upper chamber with 100 μL medium without FBS in a 24-well plate (8×10^5^ or 10×10^5^ cells/well). 600 μL medium supplemented with 30% FBS was added into the lower chamber. Cells were incubated for 40-48 h at 37°C. Finally, we fixed the cells by 4% formaldehyde and stained by Giemsa. The migration ability of cells was analyzed. Experiment was repeated in three wells.

### Wound healing assay

Lentivirus transfected cells (5×10^4^ cells/well) were plated into a 96-well dish for culturing. Scratches were made by a 96 wounding replicator (VP scientific, San Diego, CA, USA) across the cell layer with 90% confluence. RPMI-1640 medium with 0.5% FBS was added for culturing. Photographs were taken by a fluorescence microscope at 0 h, 16 h and 24 h after scratching. Cell migration rates of each group were calculated.

### RNA screening analysis

The detection of gene expression profile based on Eca-109 cells with shMEX3A or shCtrl by RNA screening analysis was completed by Shanghai Bioscienceres, Co., Ltd. (Shanghai, China). Briefly, total RNA from Eca-109 cells was extracted by the RNeasy kit (Sigma, St. Louis, MO, USA). Total RNA quality was evaluated by Agilent RNA 6000 Nano Kit on Agilent 2100 Bioanalyzer (Agilent, Santa Clara, CA, USA) and quantified using Thremo Nanodrop 2000 (Waltham, MA, USA). RNA sequencing was performed human GeneChip primeview (Affymetrix, Santa Clara, CA, USA) according to the manufacturer’s instruction and the outcomes were scanned by GeneChip Scanner Scanner 3000 (Affymetrix, Santa Clara, CA, USA). Raw data statistical significance assessment was accomplished using a Welch t-test with Benjamini-Hochberg FDR (FDR < 0.05 as significant). Ingenuity Pathway Analysis (IPA) (Qiagen, Hilden, Germany) was executed for all significant differentially expressed genes. |Z - score| > 2 is considered meaningful.

### Human apoptosis antibody array

Detection of related genes in human apoptosis signaling pathway was performed using Human Apoptosis Antibody Array (R&D Systems, Minneapolis, MN, USA) following the manufacturer’s instructions. Briefly, lentivirus transfected Eca-109 cells were collected and washed and then lysed by lysis buffer and total protein was extracted. Protein samples (0.5 mg/mL) were incubated with blocked array antibody membrane overnight at 4°C. After washing, 1:100 Detection Antibody Cocktail was added incubating for 1 hour, followed by incubated with HRP linked streptavidin conjugate for 1 h. All spots were visualized by enhanced chemiluminescence (ECL) (Amersham, Chicago, IL, USA) and the signal densities were analyzed with ImageJ software (National Institute of Health, Bethesda, MD, USA).

### Animal experiments and bioluminescence imaging

In order to further verify the effects of MEX3A in ESCC, Eca-109 cells transfected with shMEX3A or shCtrl were subcutaneously injected into each 4 week-old nude mice (4 × 10^6^ per mice, five mice in each group). We purchased these female BALB/c nude mice from Shanghai Lingchang Experimental Animals Co., Ltd (Shanghai, China). Mice’s weight and the tumor sizes were recorded two times per week using a caliper. The xenograft volume was calculated as π/6×L×W^2^ (W, width at the widest point; L, perpendicular width). Tumor burden was assessed weekly by bioluminescence imaging with IVIS Spectrum Imaging System (Perkin Elmer, Waltham, MA, USA). Mice were sacrificed 29 days post injection and tumors were extracted, imaged, and then used for Ki67 analysis. All animal studies were approved by Ethics committee of Jinling Hospital.

For Ki-67 analysis, Tumor tissues from mice were collected, after fixed and embedded with formalin and paraffin, 2 μm slides were cut and immersed in xylene and 100% ethanol for deparaffinization and rehydration. All slides were blocked with PBS-H_2_O_2_. We incubated the slides with primary antibody Ki-67 at 4°C overnight, and then incubated with goat anti-rabbit IgG HRP second antibody. Finally, we stained all slides by Hematoxylin and Eosin (Baso, Zhuhai, Guangdong, China). Stained slides were examined with a microscopic at 100× and 200× objective lens.

### Statistical analyses

Graphs were made using GraphPad Prism 6.01 (Graphpad Software, La Jolla, CA, USA). Data are expressed as the mean ± SD and Student’s T-Test was used to analyze the statistical significance between the shMEX3A group and shCtrl group. For clinical data statistical analysis, MEX3A gene expression difference in ESCC cancer tissues and normal tissues revealed in immunohistochemistry analysis was analyzed by Rank Sum test. Mann-Whitney U analysis and Spearman Rank correlation analysis was used to analyze the relationship between MEX3A expression and patients’ tumor characteristics. All statistical analysis was performed using SPSS 17.0 (IBM, SPSS, Chicago, IL, USA) and *P* < 0.05 was considered statistically significant.

## Supplementary Material

Supplementary Figures

Supplementary Tables
