# Supplementary material for: MEX3A is upregulated in esophageal squamous cell carcinoma (ESCC) and promotes development and progression of ESCC through targeting CDK6
Source: Aging (Albany NY). 2020 Nov 14;12(21):21091–113. doi: 10.18632/aging.103196 (PMC7695430; doi:10.18632/aging.103196)
Supplement: Supplementary Figures [file aging-12-103196-s001..pdf]

SUPPLEMENTARY FIGURES

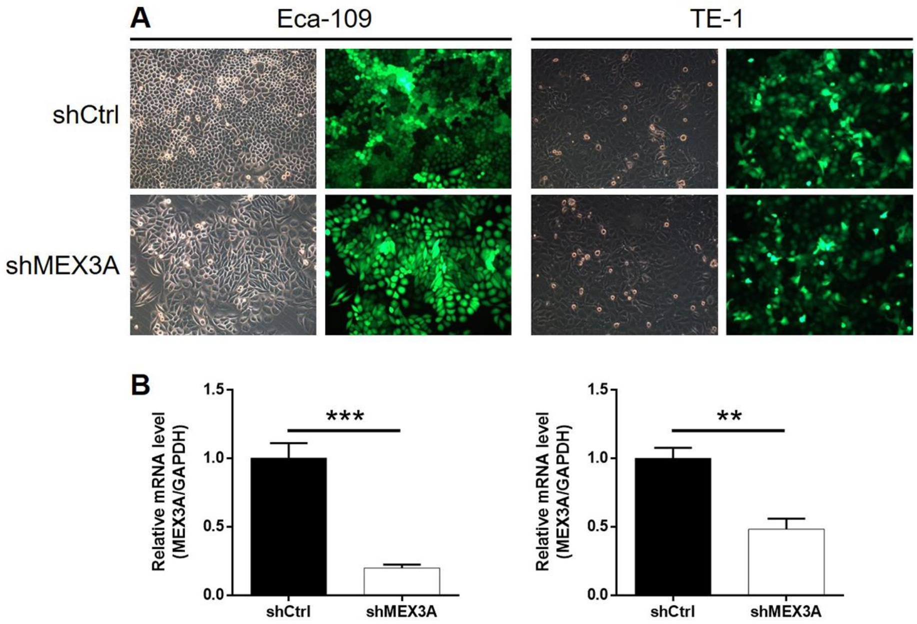

Supplementary Figure 1. (**A**) The transfection efficiencies of shMEX3A and shCtrl in Eca-109 and TE-1 cells were evaluated through observing fluorescence of GFP tag on lentivirus vector. (**B**) The efficiency of lentivirus-mediated MEX3A knockdown in Eca-109 and TE-1 cells was evaluated by qPCR. The data were expressed as mean ± SD (n ≥ 3), **P*<0.05, ***P*<0.01, ****P*<0.001.

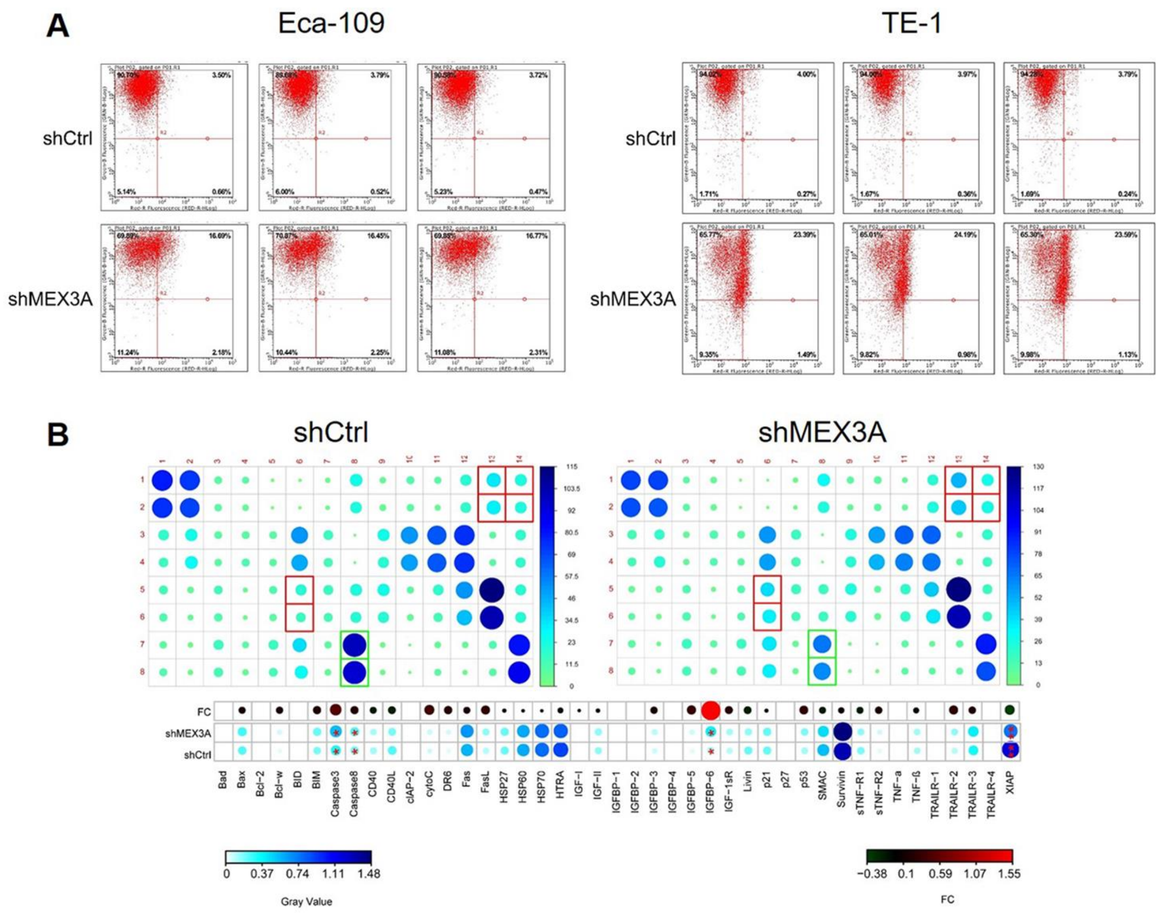

Supplementary Figure 2. (**A**) Flow cytometry was performed to show the effects of MEX3A knockdown on cell apoptosis. (**B**) Human Apoptosis Antibody Array was performed to identify the apoptosis-related proteins differentially expressed in Eca-109 cells with or without MEX3A knockdown.

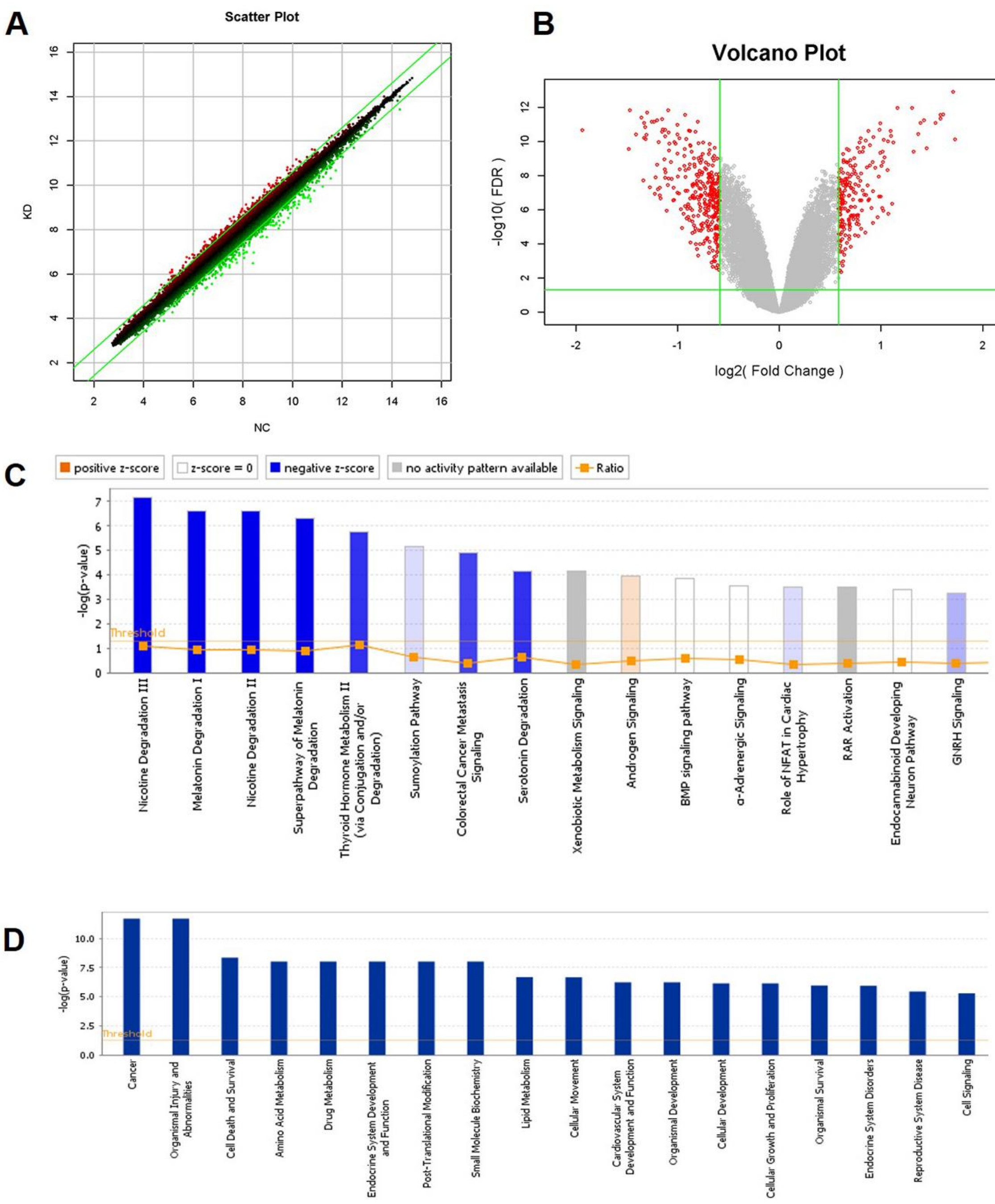

Supplementary Figure 3. (**A**, **B**) Scatter plot and volcano plot of RNA sequencing based on Eca-109 cells with or without MEX3A knockdown. In scatter plot, each point in the figure represent the signal intensity of a probe in shMEX3A and shCtrl groups; the parallel green line is the differential reference line, the red points outside the differential reference lines represent the upregulated probes in shMEX3A group, and the green points represent the down-regulated ones. In volcano plot, red dots represent the DEGs based on the threshold of absolute fold change > 1.5 and FDR < 0.05. (**C**) The enrichment of DEGs in IPA canonical signaling pathway. (**D**) The enrichment of DEGs in IPA disease & function.

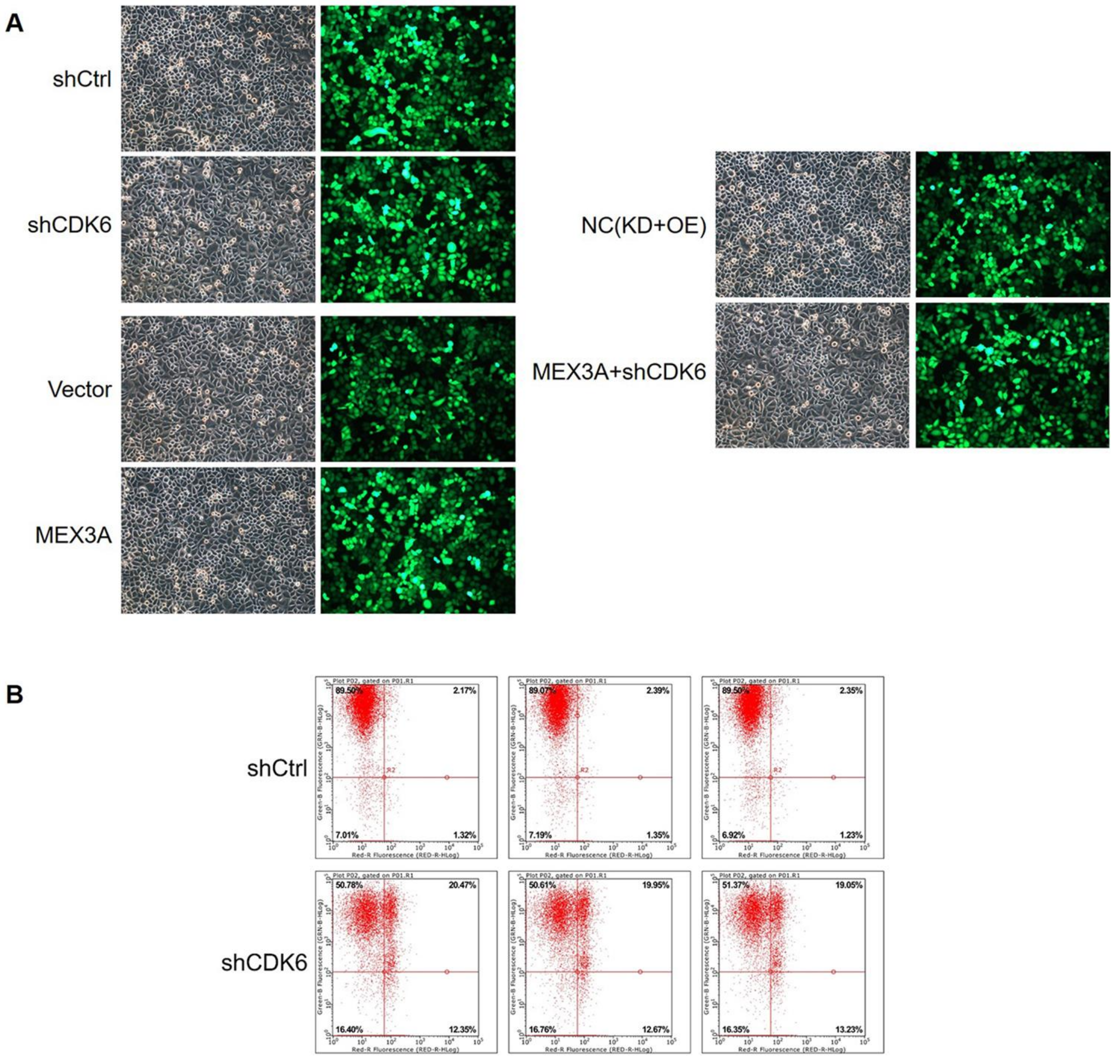

Supplementary Figure 4. (**A**) The transfection efficiencies of shCDK6, shCtrl, Vector, MEX3A (MEX3A overexpression), NC(OE+KD) and MEX3A+shCDK6 in Eca-109 cells were evaluated through observing fluorescence of GFP tag on lentivirus vector. (**B**) Flow cytometry was performed to show the effects of CDK6 knockdown on cell apoptosis.

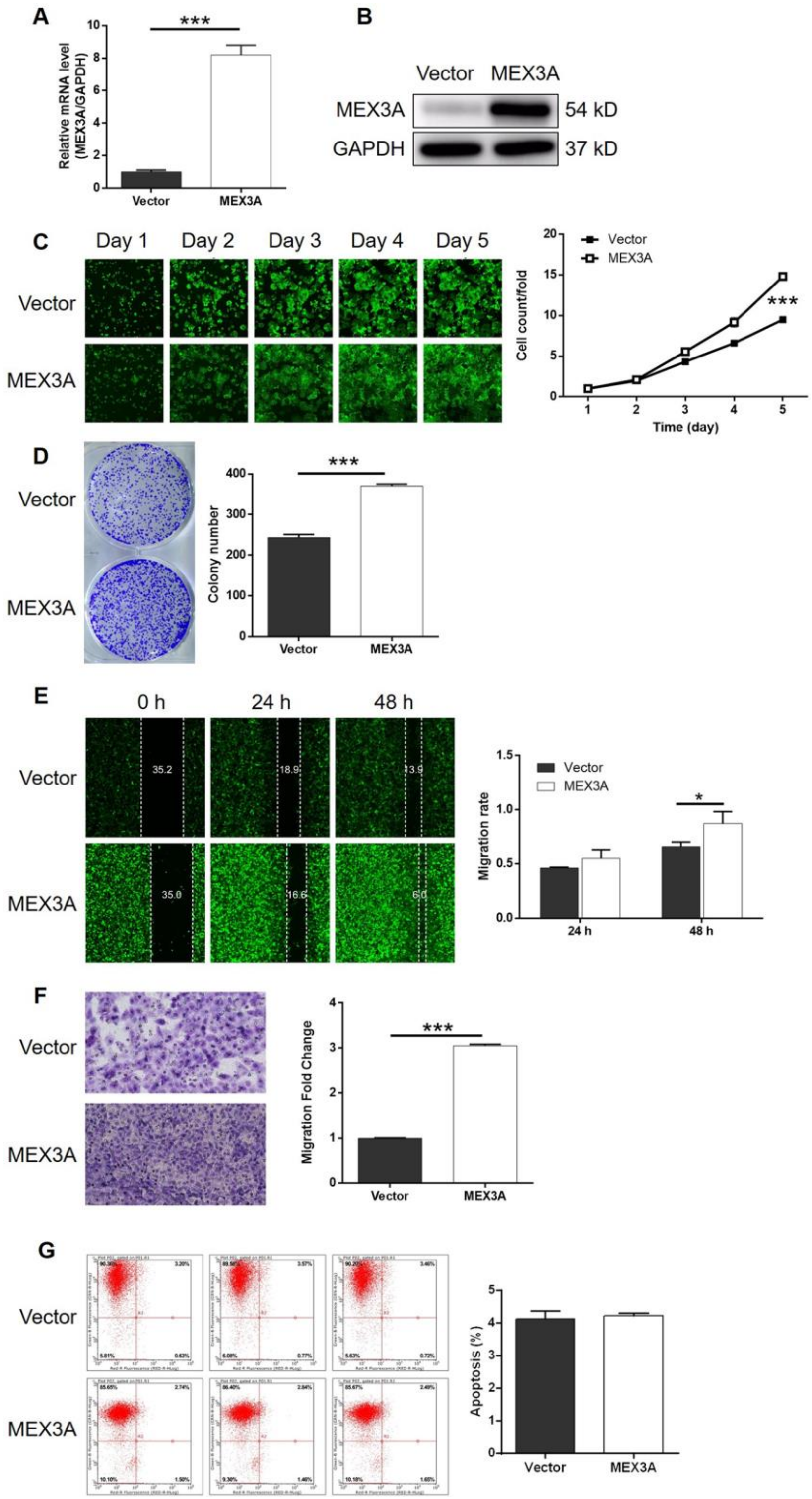

Supplementary Figure 5. Overexpression of MEX3A promoted development of ESCC *in vitro*. (**A**) The efficiency of MEX3A overexpression in Eca-109 cells was detected by qPCR. (**B**) The overexpression of MEX3A in Eca-109 cells was confirmed by western blotting. (**C**) The regulation of cell proliferation by MEX3A overexpression in Eca-109 cells was evaluated by Celigo cell counting assay. (**D**) The influence of colony formation ability of Eca-109 cells by MEX3A overexpression was examined by colony formation assay. (**E**, **F**) The effects of MEX3A overexpression on cell migration of Eca-109 cells were assessed by wound-healing (**E**) and Transwell (**F**) assays. (**G**) Flow cytometry was performed to detect cell apoptosis of Eca-109 cells with or without MEX3A overexpression. The figures are representative data from at least three independent experiments. The data were expressed as mean ± SD (n ≥ 3), *P<0.05, **P<0.01, ***P<0.001.

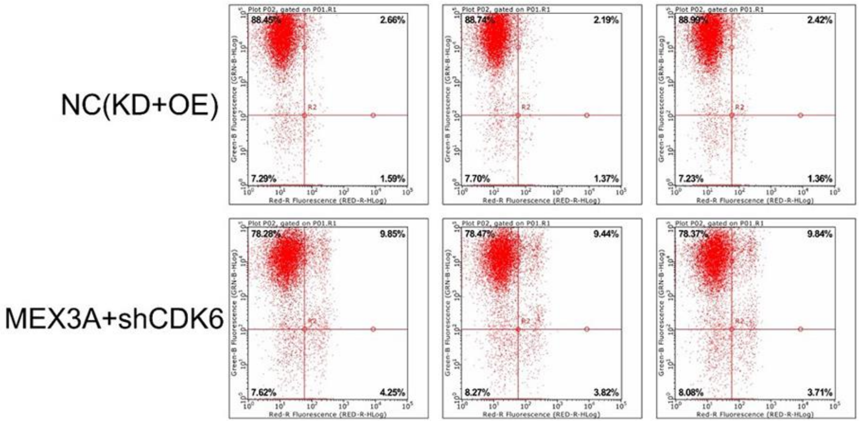

Supplementary Figure 6. Flow cytometry was performed to show the effects of MEX3A overexpression + CDK6 knockdown on cell apoptosis.
