# Supplementary material for: MEX3A is upregulated in esophageal squamous cell carcinoma (ESCC) and promotes development and progression of ESCC through targeting CDK6
Source: Aging (Albany NY). 2020 Nov 14;12(21):21091–113. doi: 10.18632/aging.103196 (PMC7695430; doi:10.18632/aging.103196)
Supplement: Supplementary Tables [file aging-12-103196-s002..pdf]

SUPPLEMENTARY TABLES

Supplementary Table 1. Primers used in qPCR.

Gene	Forward primer sequence (5'-3')	Reverse primer sequence (5'-3')
MEX3A	CGGAGTGGACTCTGGCTTTGAG	CAGAGGAGAAGAGCACGGAGGT
GAPDH	TGACTTCAACAGCGACACCCA	CACCCTGTTGCTGTAGCCAAA
UGT1A7	TGGTCGTAGTCATGCCAGAG	GGGCACTGTAGGCAGATATTCTT
RAP1A	ACGGGTTAAGGACACGGAA	TGCCAACTACTCGCTCATCTT
CDK6	TCCCAGGCAGGCTTTTCAT	GGGCACTGTAGGCAGATATTCTT
UGT1A5	GCTGGGTCACACTCAATCGT	AGGGCCTCATTATGCAGTAGC
CYP1A1	CCCTATTCTTCGCTACCTACCC	TCTGTGATGTCCCGGATGTG
NOTCH2	TGTGAGGGAGACATCAACGAG	TAAAGGCACTACGGCAAACAC
UGT1A6	TTCCTTGGACGTGATTGGTTTC	GGTCTTGGATTTGTGGGCTTT
UGT1A3	ATGCCATTTCGTGGACCCAG	TGTTCAACATTGCCATACTTCTGA
THBS1	TGTTCTCTACTGGCTTTATGTCA	GGCCTGAGCAACTCAGTCTT
CD44	TGGGTTCATAGAAGGGCACG	ATACTGGGAGGTGTTGGATGTG
UGT1A4	CTGGGCTACACTCAAGGGTT	ACAACACCTATGAAGGGCCA
UGT1A1	TGCTGGGAAGATACTGTTGATCC	GGTGTAAAATGCTCCGTCTCTG
RECQL	TAACAATGGCTGGAAAGGAGG	ATCAATGGGCAAATGACGAG
CA9	TAGCCCTGGTTTTTGGCCTC	CTAGGCTCCAGTCTCGGCTA
CYP1B1	CGACCCCCAGTCTCAATCTC	GAGTCTCTTGGCGTCGTCAG
GNB1	GCTGTTTGACCTTCGTGCTG	CAGTTGAAGTCGTCGTACCCA
MAGED1	GGGATGAGGAAGGAGATTTTGG	AGCGGGCATTCTGGTGGTAT
SDHD	GCAGCACATACACTTGTCACCG	GCAGACCCAGGAGCAAAACA
MFAP5	ACTCAAGCGACTCCAGAAACAT	TCTCATCCCAGCACTCCAAG
ARNT	TCGTGAGCAGCTTTCCACTT	CCTCATTCGGCAAATAAACG

Supplementary Table 2. Antibodies used in western blotting and IHC.

Primary antibodies	Dilution in WB	Source species	Company	Catalog No.
MEX3A	1:1000	Rabbit	abcam	ab79046
GAPDH	1:3000	Rabbit	Bioworld	AP0063
Bad	1:1000	Rabbit	abcam	ab32445
CD44	1:2000	Rabbit	abcam	ab157107
CDK6	1:1000	Rabbit	abcam	ab151247
Snail	1:1000	Rabbit	CST	3879S
Vimentin	1:2000	Rabbit	abcam	ab92547
**Primary antibodies**	**Dilution in IHC**	**Source species**	**Company**	**Catalog No.**
MEX3A	1:200	Rabbit	Abcam	ab79046
Ki-67	1:200	Rabbit	Abcam	ab16667
CDK6	1:100	Rabbit	Abcam	ab151247
**Secondary antibody**	**Dilution**		**Company**	**Catalog No.**
HRP Goat Anti-Rabbit IgG (WB)	1:3000		Beyotime	A0208
HRP Goat Anti-Rabbit IgG (IHC)	1:200		abcam	ab111909

Supplementary Table 3. Relationship between MEX3A expression and tumor characteristics in patients with ESCC analyzed by Spearman rank correlation analysis.

Tumor characteristics	index	
Lymph node positive	Pearson correlation	0.311
	Significance (two tailed)	0.003**
	n	92
Lymphatic metastasis (N)	Pearson correlation	0.280
	Significance (two tailed)	0.005**
	n	100
AJCC Stage	Pearson correlation	0.294
	Significance (two tailed)	0.003**
	n	98
